# Supplementary material for: Multiplexed imaging of nucleome architectures in single cells of mammalian tissue
Source: Nat Commun. 2020 Jun 9;11:2907. doi: 10.1038/s41467-020-16732-5 (PMC7283333; doi:10.1038/s41467-020-16732-5)
Supplement: Supplementary file 1 — Supplementary Information [file 41467_2020_16732_MOESM1_ESM.pdf]

Supplementary Information

Multiplexed imaging of nucleome architectures in single cells of mammalian tissue

Liu *et al.*

Supplementary Figures

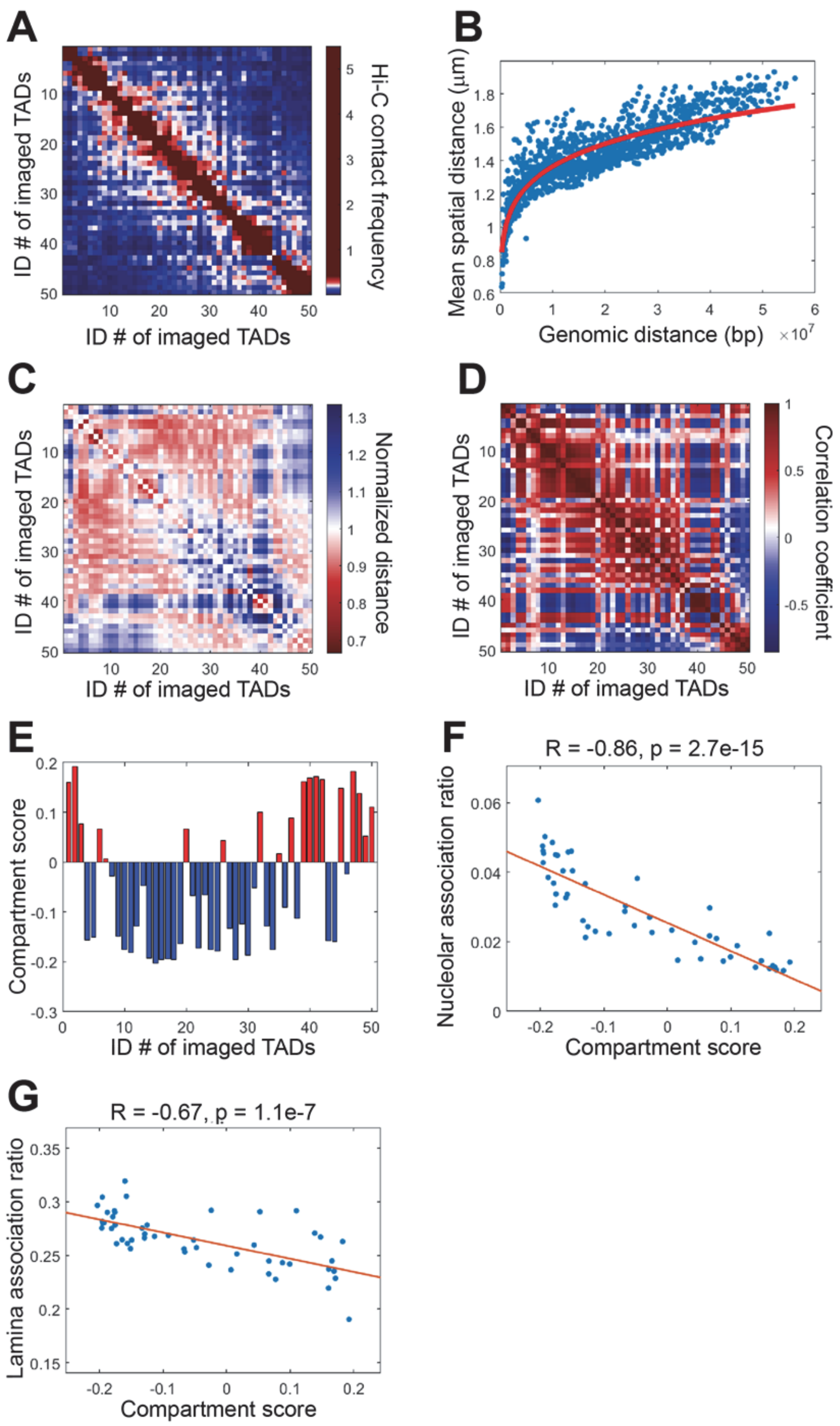

Supplementary Figure 1. Validation of MINA measurements. (**A**) Hi-C contact frequency between all pairs of the 50 imaged TADs. The Hi-C contact frequency between each pair of

TADs was calculated as the Hi-C counts between the two TADs divided by the genomic lengths of the two TADs. Raw Hi-C data were downloaded from http://chromosome.sdsc.edu/mouse/hi-c/download.html. (**B**) Mean spatial distance versus the genomic distance for all pairs of TADs. Each dot represents a pair of TADs. The red line is a power-law function fit showing the expected spatial distance at each genomic distance. (**C**) Normalized spatial distance matrix. The normalized spatial distance is calculated as the mean spatial distance divided by the expected spatial distance from B. (**D**) Pearson correlation matrix of the 50 TADs calculated from the normalized distance matrix in C. Each matrix element shows the correlation coefficient between a pair of rows (or columns) in C. (**E**) Compartment assignments of TADs based on the compartment scores. The compartment scores are the coefficients of the first principal component from a principal component analysis of the Pearson correlation matrix. Red bars (positive scores): TADs in compartment A. Blue bars (negative scores): TADs in compartment B. (**F**) Correlation between nucleolar association ratios and compartment scores. (**G**) Correlation between lamina association ratios and compartment scores. In F and G, each dot represents a TAD, and the lines show linear regression fits. The p values were calculated for Pearson's correlation using a two-sided Student's t distribution. No adjustment was made for multiple comparisons. Source data are provided as a Source Data file.

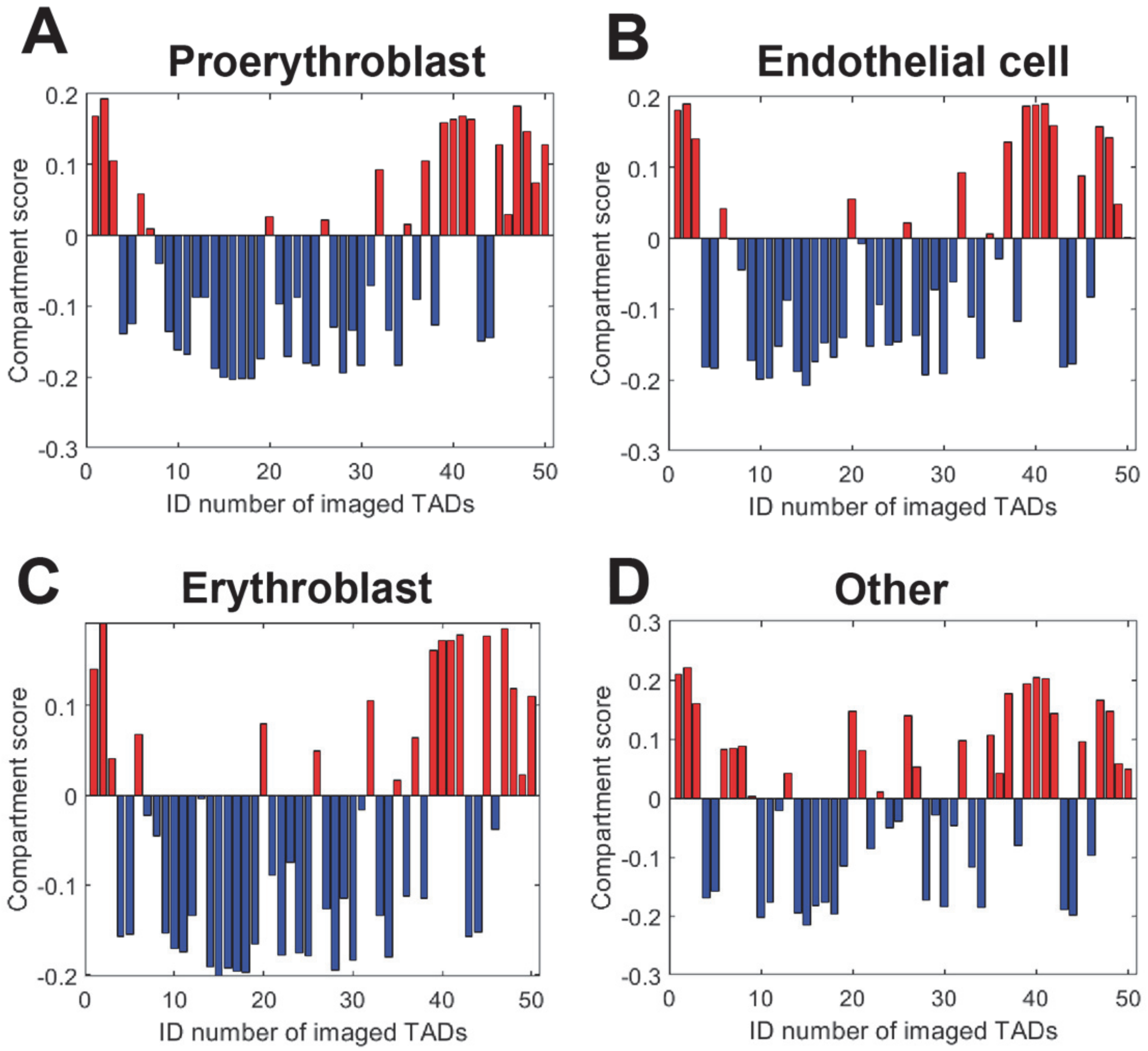

Supplementary Figure 2. Cell-type-specific compartment analyses. Compartment assignments of TADs based on their compartment scores are plotted for proerythroblasts (**A**), endothelial cells (**B**), erythroblasts (**C**), and cell type "other" (**D**). Compartment assignments of TADs in hepatocytes, megakaryocytes, and macrophages are plotted in Figure 3B. Red bars (positive scores): TADs in compartment A. Blue bars (negative scores): TADs in compartment B. Source data are provided as a Source Data file.

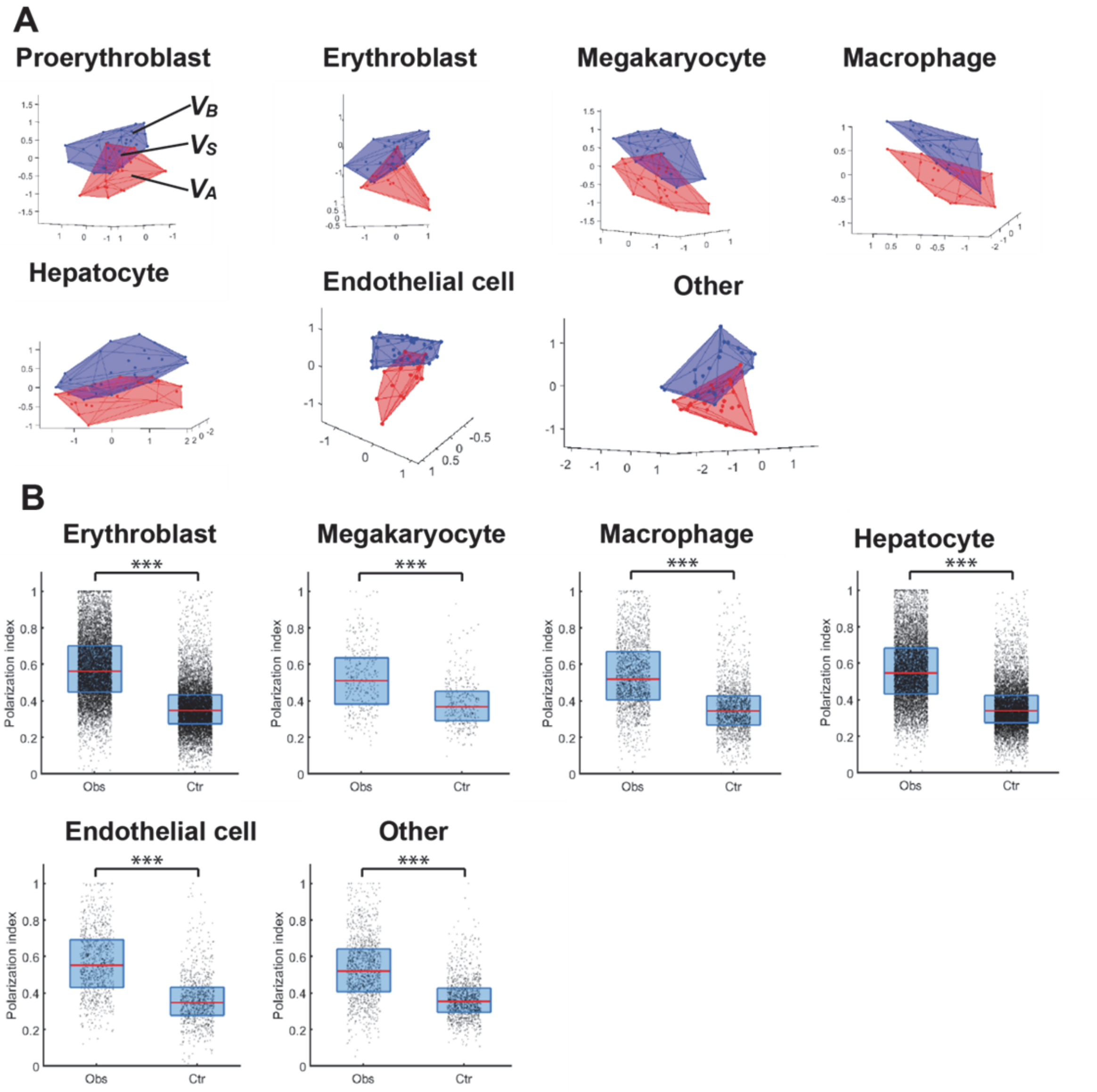

Supplementary Figure 3. Polarized organization of compartments A and B in different cell types. (**A**) 3D convex hull plots of individual chromosomes from different cell types. The blue and red shapes represent the 3D convex hulls of compartments B and A, respectively. The blue and red dots represent the 3D positions of TADs in compartments B and A. The volumes of the two convex hulls are V_B and V_A, respectively. The shared volume between the two hulls is V_S. The polarization index is defined as $\sqrt{(1 - V_S/V_A)(1 - V_S/V_B)}$. (**B**) Observed polarization indices of individual chromosomes (Obs) in comparison with those of a randomization control (Ctr), in which we randomized compartment assignments of TADs while maintaining the total number of TADs in each compartment. The dots, red lines, and blue boxes represent the values

for individual chromosomes, the median value, and the 25% – 75% quantiles. ***: p = 0 (erythroblast), 8.2e-28 (megakaryocyte), 4.0e-193 (macrophage), 0 (hepatocyte), 4.4e-120 (endothelial cell), 1.2e-128 (Other). All p values were calculated using a two-sided Wilcoxon rank sum test. “p = 0” indicates that the exact p value is smaller than the smallest positive double precision floating-point number in MATLAB (1e-307). A total of 8469, 357, 1753, 7484, 900, 1177 chromosomes were measured respectively in erythroblast (Obs median = 0.56; Ctr median = 0.35), megakaryocyte (Obs median = 0.51; Ctr median = 0.37), macrophage (Obs median = 0.52; Ctr median = 0.35), hepatocyte (Obs median = 0.55; Ctr median = 0.34), endothelial cell (Obs median = 0.55; Ctr median = 0.35) and others (Obs median = 0.52; Ctr median = 0.35). The plot for proerythroblasts is shown in Figure 3E. Source data are provided as a Source Data file.

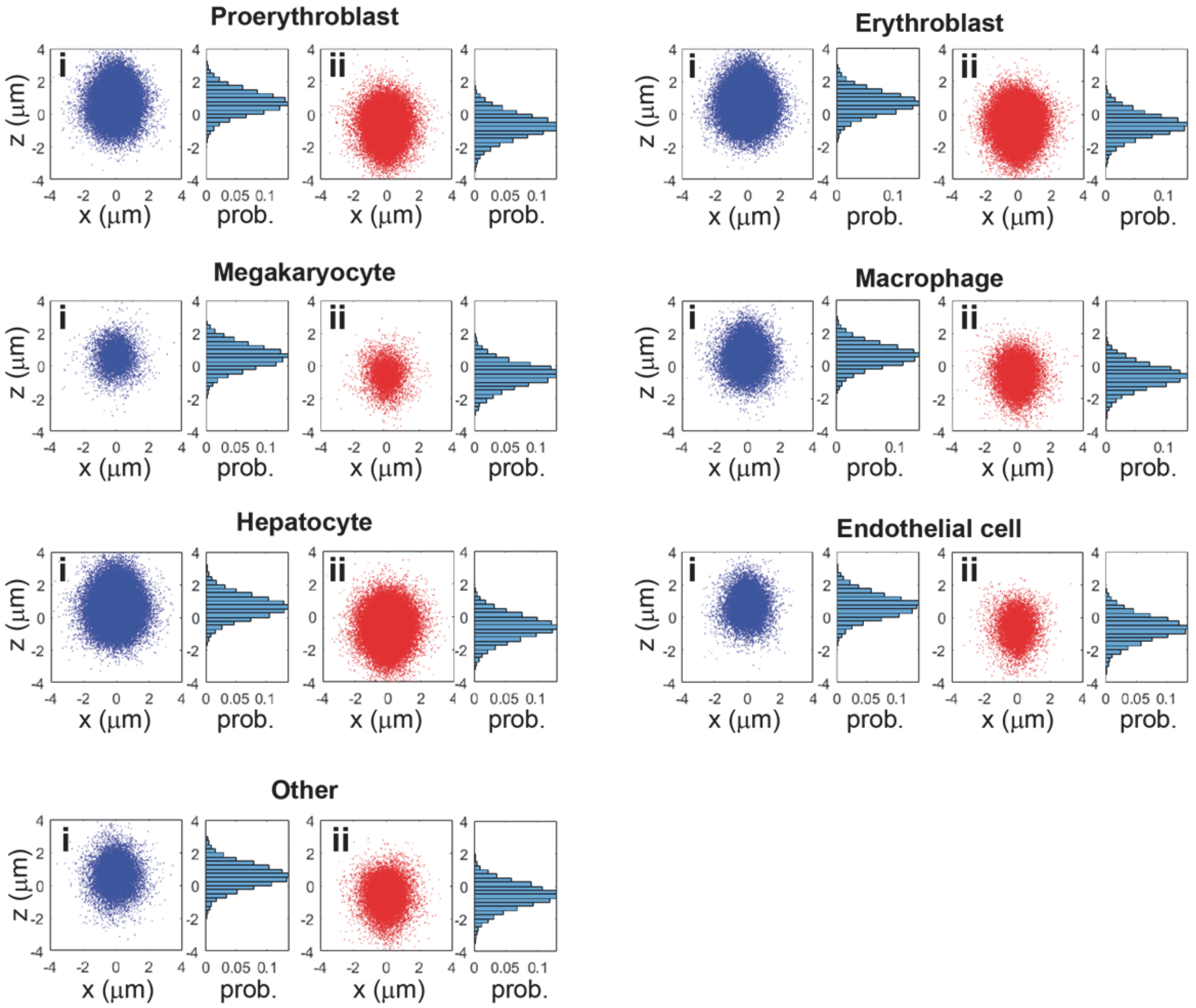

Supplementary Figure 4. Spatial distribution of compartment-A TADs relative to the center of compartment B and the spatial distribution of compartment-B TADs relative to the center of compartment A along the polarization axis. We aligned all chromosome traces of each cell type by rigidly rotating the traces so that the polarization axis of each trace, defined as a vector pointing from the centroid of compartment A to the centroid of compartment B, is aligned with the z axis. We then aligned the compartment-A centroid of each trace at the origin, and determined the distribution of compartment-B TADs in the x-z plane (i). Alternatively, we aligned the compartment-B centroid of each trace at the origin, and determined the distribution of compartment-A TADs in the x-z plane (ii). Source data are provided as a Source Data file.

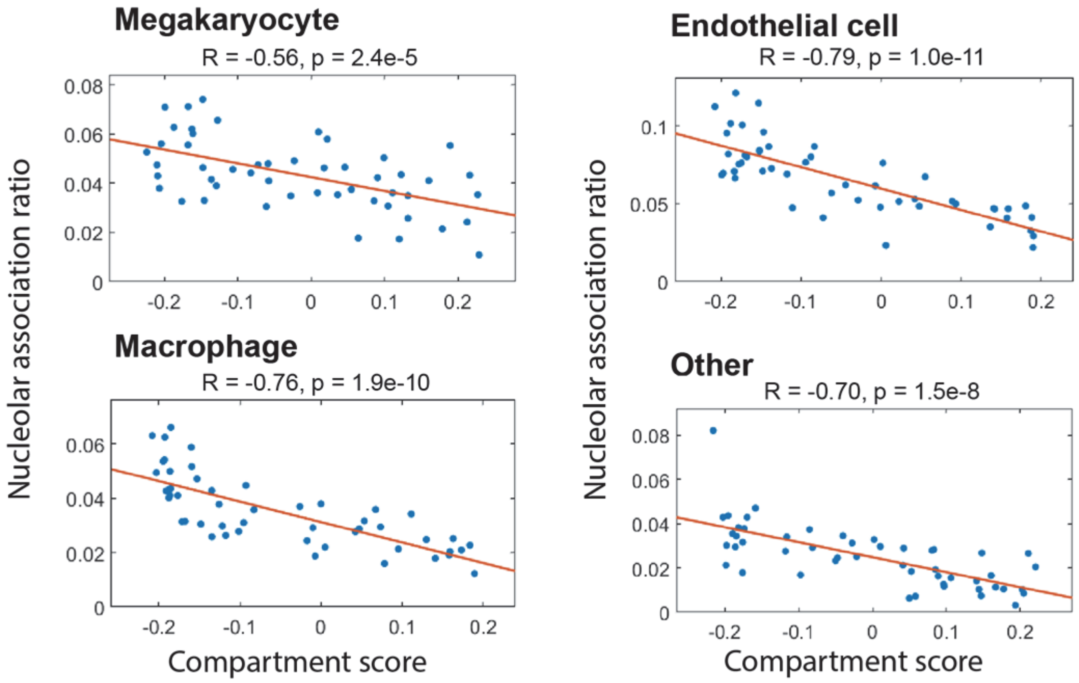

Supplementary Figure 5. Correlation between nucleolar association ratios and compartment scores in different cell types. Each dot represents a TAD. The lines show linear regression fits. The plots for hepatocytes, erythroblasts, and proerythroblasts are shown in Figure 4A. The p values were calculated for Pearson's correlation using a two-sided Student's t distribution. No adjustment was made for multiple comparisons. Source data are provided as a Source Data file.

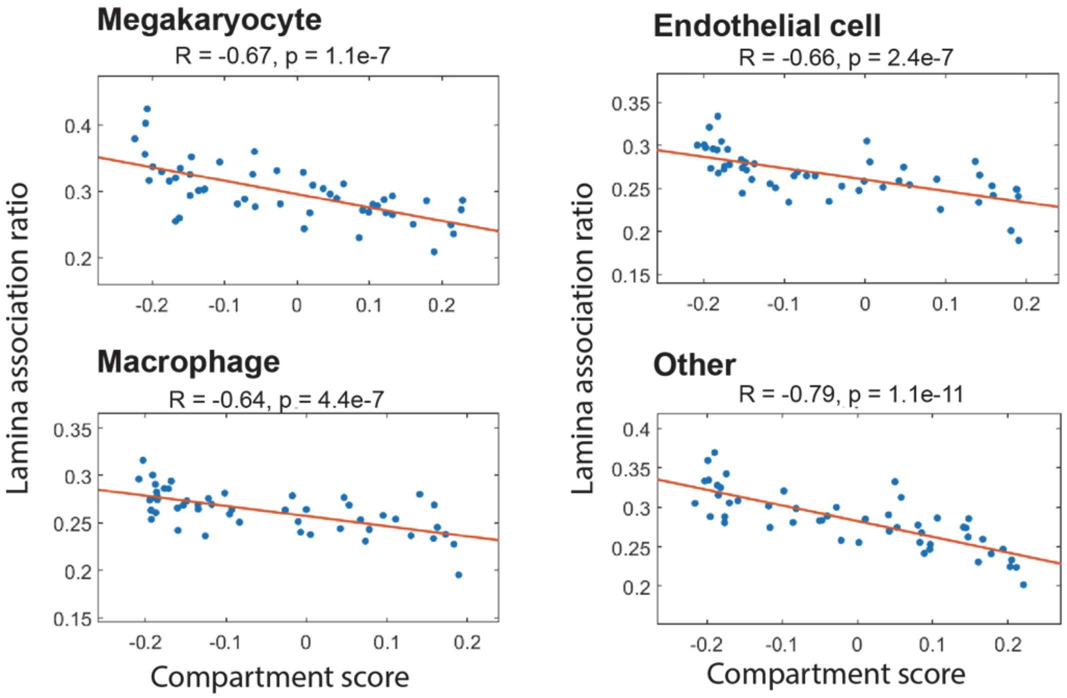

Supplementary Figure 6. Correlation between lamina association ratios and compartment scores in different cell types. Each dot represents a TAD. The lines show linear regression fits. The plots for hepatocytes, erythroblasts, and proerythroblasts are shown in Figure 4B. The p values were calculated for Pearson's correlation using a two-sided Student's t distribution. No adjustment was made for multiple comparisons. Source data are provided as a Source Data file.

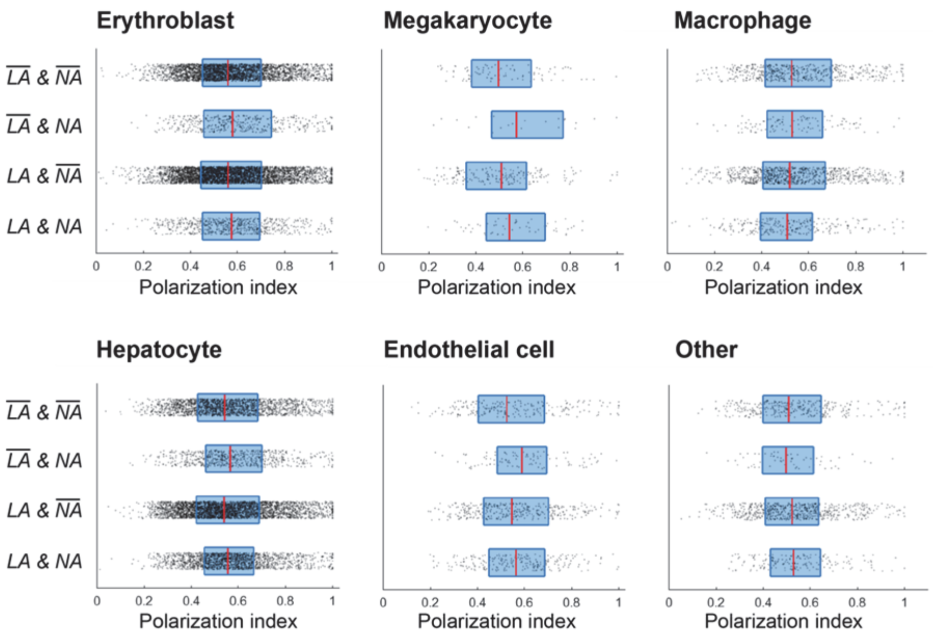

Supplementary Figure 7. Polarization indices for chromosomes with or without nucleolar or lamina association (with compartment B) in different cell types. $\overline{LA}$ & $\overline{NA}$: with neither lamina nor nucleolar association (n = 3135, 95, 532, 1897, 219, 320 chromosomes respectively in erythroblast, megakaryocyte, macrophage, hepatocyte, endothelial cell and others). $\overline{LA}$ & NA: without lamina association but with nucleolar association (n = 432, 23, 114, 632, 102, 62 chromosomes respectively in erythroblast, megakaryocyte, macrophage, hepatocyte, endothelial cell and others). LA & $\overline{NA}$: with lamina association but without nucleolar association (n = 4275, 182, 842, 3298, 347, 627 chromosomes respectively in erythroblast, megakaryocyte, macrophage, hepatocyte, endothelial cell and others). LA & NA: with both lamina and nucleolar associations (n = 627, 57, 266, 1657, 232, 168 chromosomes respectively in erythroblast, megakaryocyte, macrophage, hepatocyte, endothelial cell and others). The dots, red lines, and blue boxes represent the values for individual chromosomes, the median value, and the 25% – 75% quantiles, respectively. The plots for proerythroblasts are shown in Figure 4D. In erythroblasts, median values of the four groups (top to bottom) are 0.56, 0.58, 0.56, 0.58. In megakaryocytes, median values are 0.50, 0.57, 0.51, 0.54. In macrophages, median values are 0.53, 0.53, 0.52, 0.51. In hepatocytes, median values are 0.54, 0.57, 0.54, 0.56. In endothelial cells, median values are 0.52, 0.59, 0.55, 0.56. In others, median values are 0.51, 0.50, 0.52, 0.53. Source data are provided as a Source Data file.
