# Supplementary material for: Multiplexed imaging of nucleome architectures in single cells of mammalian tissue
Source: Nat Commun. 2020 Jun 9;11:2907. doi: 10.1038/s41467-020-16732-5 (PMC7283333; doi:10.1038/s41467-020-16732-5)
Supplement: Supplementary file 3 — Description of Additional Supplementary Files [file 41467_2020_16732_MOESM3_ESM.pdf]

Description of Additional Supplementary Files

File Name: Supplementary Data 1
Description: Genomic coordinates of labeled regions used in chromatin tracing of 50 TADs on Chr19.

File Name: Supplementary Data 2
Description: Genomic coordinates of labeled regions used in chromatin tracing of 19 5-kb loci upstream of Scd2.

File Name: Supplementary Data 3
Description: Codebook of genes targeted by RNA MERFISH.

File Name: Supplementary Data 4
Description: Template oligonucleotide sequences for chromatin tracing and RNA MERFISH.

File Name: Supplementary Data 5
Description: Sequences of PCR primers and reverse transcription primers for primary probe library syntheses.

File Name: Supplementary Data 6
Description: Sequences of dye-labeled secondary probes for chromatin tracing and RNA MERFISH.
