# Supplementary material for: Multiplexed imaging of nucleome architectures in single cells of mammalian tissue
Source: Nat Commun. 2020 Jun 9;11:2907. doi: 10.1038/s41467-020-16732-5 (PMC7283333; doi:10.1038/s41467-020-16732-5)
Supplement: Supplementary file 10 — Reporting Summary [file 41467_2020_16732_MOESM10_ESM.pdf]

nature research

Corresponding author(s): Siyuan Wang
Last updated by author(s): May 12, 2020

Reporting Summary

Nature Research wishes to improve the reproducibility of the work that we publish. This form provides structure for consistency and transparency in reporting. For further information on Nature Research policies, see our Editorial Policies and the Editorial Policy Checklist.

Statistics

For all statistical analyses, confirm that the following items are present in the figure legend, table legend, main text, or Methods section.

n/a	Confirmed	
☐	☒	The exact sample size (*n*) for each experimental group/condition, given as a discrete number and unit of measurement
☐	☒	A statement on whether measurements were taken from distinct samples or whether the same sample was measured repeatedly
☐	☒	The statistical test(s) used AND whether they are one- or two-sided<br>*Only common tests should be described solely by name; describe more complex techniques in the Methods section.*
☐	☒	A description of all covariates tested
☒	☐	A description of any assumptions or corrections, such as tests of normality and adjustment for multiple comparisons
☐	☒	A full description of the statistical parameters including central tendency (e.g. means) or other basic estimates (e.g. regression coefficient) AND variation (e.g. standard deviation) or associated estimates of uncertainty (e.g. confidence intervals)
☐	☒	For null hypothesis testing, the test statistic (e.g. *F*, *t*, *r*) with confidence intervals, effect sizes, degrees of freedom and *P* value noted<br>*Give P values as exact values whenever suitable.*
☒	☐	For Bayesian analysis, information on the choice of priors and Markov chain Monte Carlo settings
☒	☐	For hierarchical and complex designs, identification of the appropriate level for tests and full reporting of outcomes
☐	☒	Estimates of effect sizes (e.g. Cohen's *d*, Pearson's *r*), indicating how they were calculated

Our web collection on statistics for biologists contains articles on many of the points above.

Software and code

Policy information about availability of computer code

Data collection	Data were collected using open source python codes from https://github.com/ZhuangLab/storm-control. No other software is used for data collection.
Data analysis	The MATLAB programs for data analysis and simulation are available at https://github.com/SiyuanWangLab/MINA. The following softwares are used in this study: OligoArray2.1, BLAST-2.9.0+, and MATLAB version R2018a. Metropolis algorithm was implemented using MATLAB version R2018a.

For manuscripts utilizing custom algorithms or software that are central to the research but not yet described in published literature, software must be made available to editors and reviewers. We strongly encourage code deposition in a community repository (e.g. GitHub). See the Nature Research guidelines for submitting code & software for further information.

Data

Policy information about availability of data

All manuscripts must include a data availability statement. This statement should provide the following information, where applicable:

- Accession codes, unique identifiers, or web links for publicly available datasets
- A list of figures that have associated raw data
- A description of any restrictions on data availability

The genomic positions of TADs profiled in large-scale chromatin tracing were downloaded from http://chromosome.sdsc.edu/mouse/hi-c/download.html. Mouse E14.5 fetal liver Hi-C data were downloaded from GSM1718024 [https://www.ncbi.nlm.nih.gov/geo/query/acc.cgi?acc=GSM1718024]. All chromatin traces, RNA profiles, lamina/nucleolar association data, and MATLAB programs for data analysis and simulation are available at https://campuspress.yale.edu/wanglab/MINA/. Raw imaging data have not been deposited in a public repository because of the prohibitively large volume, but are available from the corresponding author on

request. Bulk RNA sequencing data from this work are available at GSE148072 [https://www.ncbi.nlm.nih.gov/geo/query/acc.cgi?acc=GSE148072]. The source data underlying Figures 1-6 and Supplementary Figures 1-7 are provided as a Source Data file.

Field-specific reporting

Please select the one below that is the best fit for your research. If you are not sure, read the appropriate sections before making your selection.

☒ Life sciences ☐ Behavioural & social sciences ☐ Ecological, evolutionary & environmental sciences

For a reference copy of the document with all sections, see nature.com/documents/nr-reporting-summary-flat.pdf

Life sciences study design

All studies must disclose on these points even when the disclosure is negative.

Sample size	Our sample sizes for different cell types are: Proerythroblast: N = 4873. Megakaryocyte: N = 358. Macrophage: N = 1773. Hepatocyte: N = 7543. Erythroblast: N = 8525. Endothelial Cell: N = 911. Other: N = 1181. Sample sizes were counted from images. Sample sizes are large enough as all conclusions were reproducible in each replicate.
Data exclusions	When analyzing single-cell RNA data, we excluded "cells" larger than 20,000 pixels in area as these were usually empty regions in tissue sections. We excluded "cells" smaller than 2,500 pixels in area as these were usually non-cell particles. We excluded cells with less than 10 detected RNA molecules to ensure high quality in the cell type identification analyses. These exact exclusion criteria were not pre-established but the concepts of these exclusion criteria are similar to those generally used in previous single cell RNA sequencing analyses.
Replication	Measurements were from four biological replicates. All four replicates contain measurements of the TAD-to-chromosome scale chromatin folding of chromosome 19, lamina and nucleolar associations, and single-cell RNA profiles. Two of the four replicates also contain measurements of the fine-scale chromatin folding of the cis-regulatory region of Scd2. All measurements were reproducible in each replicate.
Randomization	Mouse E14.5 fetal livers were randomly selected for data collection. Fetal liver tissue areas were selected randomly during imaging. All experimental data were from the randomly selected fetal liver tissue.
Blinding	No blinding was used since no comparison was made between experimental groups.

Reporting for specific materials, systems and methods

We require information from authors about some types of materials, experimental systems and methods used in many studies. Here, indicate whether each material, system or method listed is relevant to your study. If you are not sure if a list item applies to your research, read the appropriate section before selecting a response.

Materials & experimental systems

n/a	Involved in the study	
☐	☒	Antibodies
☒	☐	Eukaryotic cell lines
☒	☐	Palaeontology and archaeology
☐	☒	Animals and other organisms
☒	☐	Human research participants
☒	☐	Clinical data
☒	☐	Dual use research of concern

Methods

n/a	Involved in the study	
☒	☐	ChIP-seq
☒	☐	Flow cytometry
☒	☐	MRI-based neuroimaging

Antibodies

Antibodies used	anti-fibrillarin primary antibody (Abcam Cat# ab5821); Alexa Fluor 647-conjugated anti-rabbit secondary antibody (Molecular Probes Cat# A-31573)
Validation	The anti-fibrillarin antibody has been tested for specificity by the manufacturer through western blot and blocking by immunising peptide.

Animals and other organisms

Policy information about studies involving animals; ARRIVE guidelines recommended for reporting animal research

Laboratory animals	Pregnant female C57BL/6 mice at the age of 8-15 weeks from the Jackson Laboratory were used for all experiments. All mice were maintained under 12h light/12h darkness cycles with constant conditions of temperature (22°C) and humidity (40%-60%).
Wild animals	The study did not involve wild animals.

Field-collected samples	The study did not involve samples collected from the field.
Ethics oversight	All procedures have been approved by the Institutional Animal Care and Use Committee of Yale University.

Note that full information on the approval of the study protocol must also be provided in the manuscript.
